# Plant Bridge: Connecting Separated Objects Using Plant Growth

**DOI:** 10.3390/biomimetics10050321

**Published:** 2025-05-15

**Authors:** Kodai Ochi, Mitsuharu Matsumoto

**Affiliations:** Department of Informatics, The University of Electro-Communications, Chofu 182-8585, Japan; o2340004@edu.cc.uec.ac.jp

**Keywords:** plant growth, plant bridge, bio-hybrid

## Abstract

In recent years, there has been development in bio-hybrid actuators that utilize living organisms themselves, as opposed to biomimetics. However, most of the plants and animals used for these purposes are no longer actually alive, as their corpses, parts, or seeds are used. There is research on the use of microorganisms, but it is limited to use in building materials. Here, we focused on plants in terms of their ease of growth with water and light and their ability to change shape significantly from seed through growth. Therefore, we propose a material that incorporates living plants. The objective of this research is to realize the shape change of this material by using the property of plants to grow toward light. In the experiment, we confirmed that plants growing from two devices cross-linked between the devices by controlling the direction of growth using peas. The bridged plants did not break when a mass of up to 575 g was placed on it and indicated a load-bearing capacity of more than 6.6 times from the mass ratio. Then, it is demonstrated that the robot could cross over that.

## 1. Introduction

In recent years, there has been a lot of research on biomimetic technology. Some examples are robots that imitate geckos that can climb walls and robots that imitate snakes that can move robustly [1,2,3,4]. In addition, worm-type robots and starfish-type robots, which were developed as biomimetic soft robots, are more biological [5,6,7]. However, these are imitations, so they are different from the organisms themselves. Therefore, the biomimetic may be inferior to the performance of the organism or miss other properties that the organism possesses.

Recently, bio-hybrid actuators have been developed that do not imitate living organisms but use the organisms themselves. For example, the pneumatic gripper using spiders made use of actual spider corpses [8]. Finger-shaped robots using living cells as skins have also been reported [9,10]. In addition, there is some research that uses plants as well as living organisms. For example, the electrically controllable gripper using flycatcher leaves has been reported [11]. There is also the paper-like soft actuator using pollen, the pinecone robot, and the awn seed robot that are activated by dry/wet control [12,13,14,15]. Since these studies use the animals and plants themselves, it is possible to use the characteristics of the animals and plants directly. However, from the viewpoint of ease of handling, most of them are of limited utilization, such as the dead bodies of living organisms or their parts, seeds, the pollen of plants, and so on.

On the other hand, in the field of architecture, the structural materials that use the metabolism of photosynthetic cyanobacteria, called living building materials, have been developed [16]. Concrete restoration technology using microorganisms has also been reported [17]. These studies differ from previous biohybrid technologies in that they use the microorganisms themselves, while they are still alive. However, these studies are in the architectural field, so they are assumed to be used in a static environment. Therefore, it is a precondition that the nutrients and building materials necessary for microorganisms’ growth can be supplied sequentially from the outside. This limits the usage situations and makes it especially difficult to use in a dynamic environment.

Furthermore, studies on living root bridges, which are structures made of plants, have been reported [18,19,20]. It is commonly found in Meghalaya, India, and consists of the well-developed aerial roots of *Ficus elastica* Roxb. ex Hornem. (Moraceae) planted on separated banks. These aerial roots are shaped into a living root bridge by the indigenous people, and the bridge becomes stronger with years of human maintenance and plant growth. Thus, these are sophisticated structures made of living plants; however, as mentioned in the above, the generation of bridges has the disadvantage of requiring many years of maintenance by human hands. On the other hand, studies on autonomous plant growth through engineering control have also been reported [21,22]. The purpose here is to assist plant growth by monitoring the plant growth environment using sensors, and to mechanically strengthen the structure by controlling the direction of growth through the tropism of plants. This is a bio-hybrid structure in which artificial materials and plants coexist in symbiosis. However, in that experiment, the main focus is on controlling plant growth on a structure fixed near the planter with climbing plants, so it is difficult to change the position of the system.

Based on the above, we have proposed a framework of plant-incorporated materials that can directly utilize the characteristics of living plants even in dynamic environments. In particular, plant growth requires water and light, which are relatively easier to access from the natural environment than artificial materials. In addition, sprouts in the early stages of germination grow quickly and change their shape and volume significantly from their seed state. Sprouts are also low cost to cultivate, because they can be grown hydroponically. Based on the above, we focused on sprouts, and in this study, we specifically utilized peas. In addition, plant growth is affected by stimuli, such as gravity and light, which determine the rate and direction of growth [23]. Therefore, we studied shape deformation technology that utilizes growth changes caused by the tropism of plants. In this study, we fabricated two devices with plant seeds and a light source and aimed to connect the two devices placed across from each other by controlling plant growth. We consider that the realization of this will allow for the connection of the separated areas by plants grown from the two devices. In this study, we have developed a device to control the direction of plant growth and have succeeded in making the connection by actually growing the plants. In addition, we confirmed the degree of connection strength with these devices and conducted an experiment in which a robot actually crossed a bridge created by the plants.

## 2. Supposed Scenario

**Figure 1:**
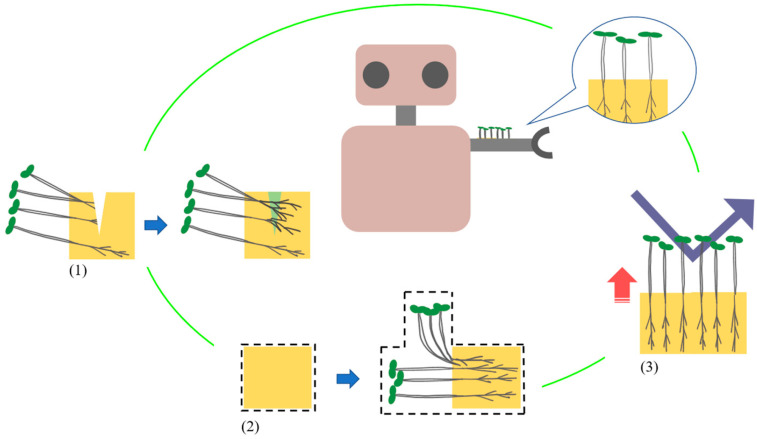
Schematic diagram of the use of plant-incorporated material as a robot skin. The following properties by plant growth: (1) self-healing, (2) shape deformation, (3) strengthening rigidity. Blue arrows represent changes, red arrows represent rigidity increases, and dark blue arrows represent external forces.

Plant-incorporated materials aim to be used in dynamic environments by utilizing living plants. We assume that dynamic environments are mainly robots and are researching the use of this material as a robot skin. Figure 1 shows a schematic diagram of the plant-incorporated material used as a robot skin. We consider that plant-incorporated materials can have three properties: (1) self-healing, (2) shape deformation, and (3) strengthening rigidity, by using plant growth. The numbers correspond to those in Figure 1. This material differs from building materials that use the metabolites of microorganisms and takes advantage of the fact that plants transform their volume and material through growth from the state of small seeds. In addition, the plants themselves are capable of regenerating damage caused by growth. Through the above, we have been investigating the use of plant sprouts for strengthening rigidity and self-healing [24,25]. In these processes, pea sprouts have been found to be useful in terms of growth rate and stiffness increase. Therefore, in this research, we developed a method for shape deformation using pea sprouts, which had not been seen before. Here, we use the phototropism property of plants for shape deformation. So, we used light to control the direction of growth and transform the plants into the intended shape. In addition, we observed that adjacent peas are often entangled with each other during the process of pea growth so far. Based on this, in this study, we conducted an experiment to connect two devices by controlling the growth shape of peas planted in the two devices and entangling their stems with each other. This indicates that two devices placed at separate locations can be cross-linked by plant growth. In addition, we envision scenarios for the use of devices connected by plants, such as bridging a robot. Generally, robots that move on the ground are stranded when their paths are separated by steps, ditches, and other obstacles. For such scenes, jumping robots or multi-agent robots that become a path for themselves are effective [26,27,28]. However, we consider that they have limited applications and are difficult to operate. In addition, supplies and the time required for installation are bottlenecks when considering actually connecting the separated areas with pathways. Therefore, we aim to make it possible for existing general purpose robots to move on a device in which two separated areas are bridged by plants.

## 3. Growth Equipment Prototype and Experiment

### 3.1. Fabrication of Equipment

**Figure 2:**
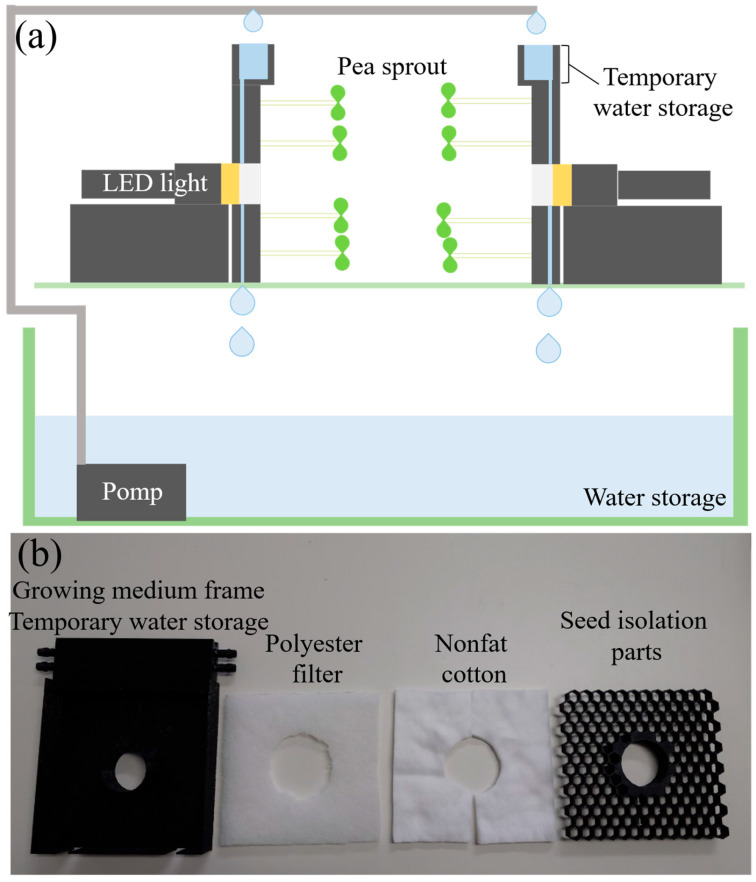
Plant hydroponics equipment for conducting cross-linking experiments using plants. (**a**) Schematic diagram of the entire cross-linked plant growth equipment. (**b**) Appearance of the material constituting the culture medium section of the plant growth device.

The objective of this study is to connect two segregated sections by using plant growth. Figure 2a is a schematic diagram of the designed plant growth experimental equipment. The device was molded in PLA resin by a 3D printer. Figure 2b shows the appearance of the growth medium device. We prepared two of these growth medium devices and grew plants with the sowing surfaces facing each other. In addition, there is a hole in the center of the device’s sowing surface, and a light source is placed behind the device, so that light is irradiated from the hole to the device across from it. The light source was an LED handy light with a nominal value of approximately 550 lm, which was modified to be power-supplied to enable constant lighting.

In the previous study, seeds were held in place by an elastic polyethylene net on the surface of the medium [29]. In this process, we observed that the seeds were biased in the direction of gravity within the medium when the medium was stood vertically. Therefore, in this equipment, seed isolation parts were installed to evenly distribute the seeds on the medium, and nets were used on top of the parts to prevent them from falling. There were a total of 152 regular hexagonal holes of about 6 mm per side in the seed isolation parts, where pea seeds were stored one by one.

Water ass transferred by gravity through small holes from a temporary water storage unit at the top of the device to the lower medium and drained from the bottom of the medium. Overflow water from the temporary water storage unit was similarly sent to the water storage tank that exists in the lower part of the device. Water was constantly circulated from the water storage tank by a pump to the growing medium device to keep the medium moist. For the culture medium, we used a 10 mm-thick nonfat cotton with high water retention properties for the lower layer and a 10 mm-thick polyester filter with high elasticity for the upper layer. The flat area of the growing medium was approximately 150 mm × 145 mm, and a hole of approximately 50 mm in diameter for the light source hole existed in the center of this area.

### 3.2. Growth Experiment

We conducted an experiment to investigate whether the fabricated equipment would allow the plants to grow and cross-link. Here, the distance between the media surfaces of the two devices was approximately 30 cm. The equipment was placed in an environment where light from the outside was blocked by a blackout curtain. The light source on the equipment was turned on about one week after the start of growth. This is because, when exposed to light immediately after germination, plant sprouts begin photosynthesis early and open their buds, which makes it difficult for them to pass through the mesh of the net. Based on the above, we allowed a period of time for the entire seed to grow spindly a little after germination.

The direction of plant growth is generally determined by gravity and light. Therefore, we considered that the plant grows as follows: In the early stages of growth, the plants grow in a direction that defies gravity, because the light source is not turned on. Later, when the light source is turned on, the plant detects the light and begins to grow in the direction of the light source.

**Figure 3:**
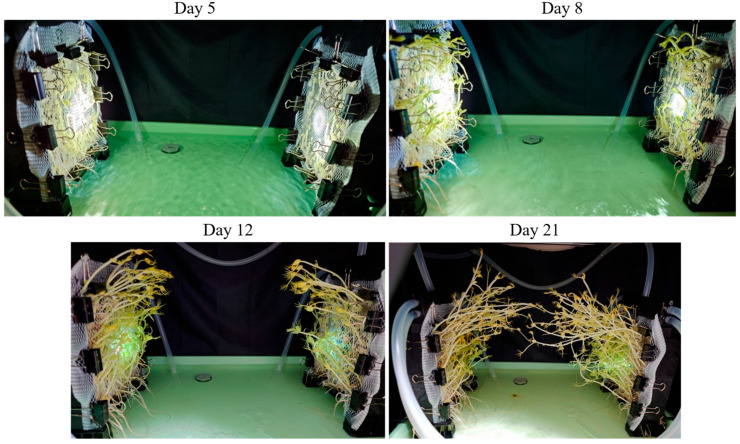
The plant growth process in the prototype cross-linked plant growth equipment. From top to bottom, the images are lined up with the time from the start of growth.

Figure 3 shows the plants during the growing period. The growing period was 28 days. We measured the temperature and humidity of the growing environment during the second half of the growing period. The average temperature was 30.9 °C, and the average humidity was 73.4%. As shown in Figure 3, at first the plants were growing upward, but by the 8th day of growth, the direction of growth was changed by light, and by the 21st day, the peas were close to each other until they were in contact with each other. However, in the experiment, no further plant growth was observed, and the device connection failed. We considered this result was caused by instability in light intensity due to contact defects in the light source mounted on the device and also by a mismatch in scale between the scale of plant growth and the distance between the devices. In addition, we thought that it was necessary to improve the germination environment for the more stable growth of the plants.

## 4. Improvement of Growth Equipment

**Figure 4:**
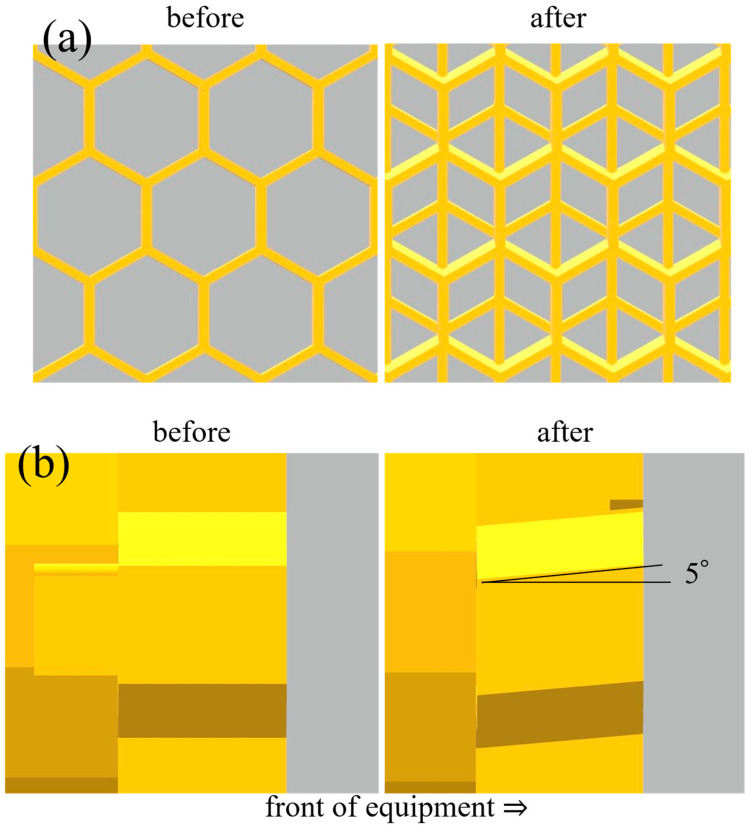
Schematic diagram of improvements for stable germination and growth of seed isolation parts in a cross-linked plant growth equipment. (**a**) The mesh structure was added. (**b**) Each segment of the seed isolation part was sloped from the horizontal.

Since the connection of the two devices due to plant growth could not be achieved in the previous experiment, we decided to improve the equipment and review the settings of the distance between the devices. In this case, first, the distance between the devices was set to about 15 cm, one-half of the distance in the previous experiment. We also modified the light source on the device to improve the contact for more stable light output. Finally, we decided to improve the parts that hold the position of the seeds in front of the medium. Figure 4 shows a schematic of the changes made to the parts for fixing the seed position. In the previous experiment, the seeds were supported by a net attached to the front of the component to prevent them from falling out of the device. However, when plant elongation after germination occurred, plant individuals were observed to grow upward between the net and the seed positioning component due to gravitropism before the buds could pass through the net meshes. Therefore, in this experiment, as shown in Figure 4a, the use of nets was discontinued, and a mesh structure was pre-installed on the seed positioning component with a mesh roughness sufficient to allow the sprouts to pass through but not the seeds. In addition, in the previous experiment, we observed seeds that did not progress to germination. This may have been due to insufficient contact with water, a condition for seed germination. As shown in Figure 4b, the seed positioning component used in the previous experiment supported the seeds on a horizontal surface, so the seeds were free to roll from the media side to the front side in the device. This is considered to be one of the reasons for the failure of the seeds to germinate. For this reason, in this experiment, the seed location fixing section was inclined at a 5-degree angle to the horizontal. This facilitated the positioning of the seeds toward the medium by gravity.

**Figure 5:**
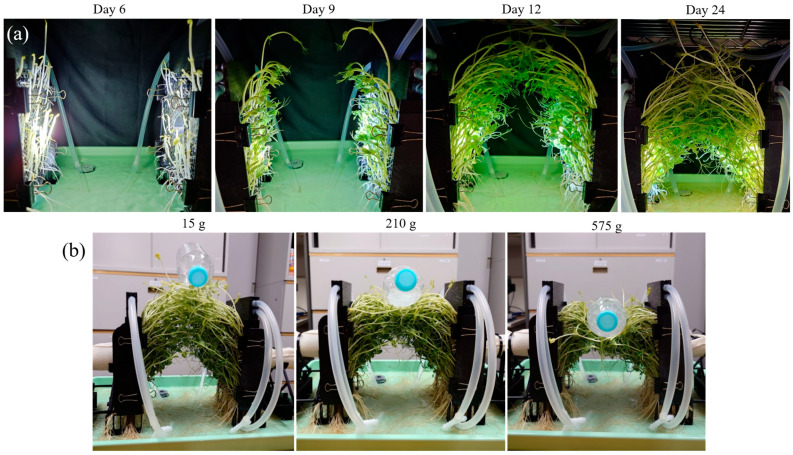
The experiment in the improved cross-linked plant growth equipment. (**a**) The process of plant growth and cross-linking. From left to right, the images are lined up with the time from the start of growth. (**b**) Connection strength experiment by placing a weight on top of the cross-linked plant. From left to right, the images are lined up with the mass of the weights increasing.

After these modifications, we grew peas and observed their growth using the same procedure as in the previous experiment. The growing period was 25 days. The average temperature and humidity of the growing environment during this period were 24.4 °C and 84.0%, respectively. Figure 5a shows the process of the growth experiment. In contrast to the previous experiment, plants can be seen to green up and open buds and leaves through sufficient photosynthesis. The amount of germination was also seen to have increased. On the 12th day of growth, sprouts from both devices began to contact each other, and the plants then grew entangled with each other. As a result, the two devices appear to be connected by the plant throughout its growth.

Based on these results, we conducted an experiment in which a weight was placed near the center on the plant between devices to confirm the connection strength of the cross-linked plants. The weights were plastic bottles filled with water and water-absorbing resin. We adjusted the mass of the weights by varying the amount of water and water-absorbing resin. Here, we decided to include the water-absorbent resin with the container, because it was difficult to place the container on the plant, as the container with water only would affect the water. The mass of the weight was increased in 5 g increments from 15 g to 575 g, the maximum container volume.

The results of the connection strength experiment are shown in Figure 5b. As shown in Figure 5b, the cross-linked plants did not break and remained connected even when the mass was increased to 575 g, the volume limit of the container. However, as the mass of the weight placed on top of the plant increased, the plant was deformed, and the center of the plant was bent.

Based on these results, we indicate the load-bearing capacity of the plant cross-linked structures. Thus, we calculated the ratio of the mass of the weights to the mass of the structure. Here, we define the mass of the plant cross-linked structure. The equipment consists of various components, such as a base to fix the medium and culture medium, water, tubes, and LED lights, in addition to the plants. However, if we assume that the devices and the culture medium were sufficiently fixed during the experiment, it is the stems and leaves of the plants that supported the weights. From the above, the mass of the structure is defined as the mass of the upper part of the stem from the base of the plant. In addition, each plant shows a different degree of growth. So, in this case, we assume that all plants planted in the equipment grew uniformly. Here, we used as a reference the mass of the plant when half the amount of seeds in this experiment were sown and grown using the same equipment. From the above, we estimated the mass of the plant cross-linked structure used in this experiment to be 87 g. Therefore, the load-bearing capacity of the cross-linked plants was at least 6.61 times greater based on the mass ratio.

**Figure 6:**
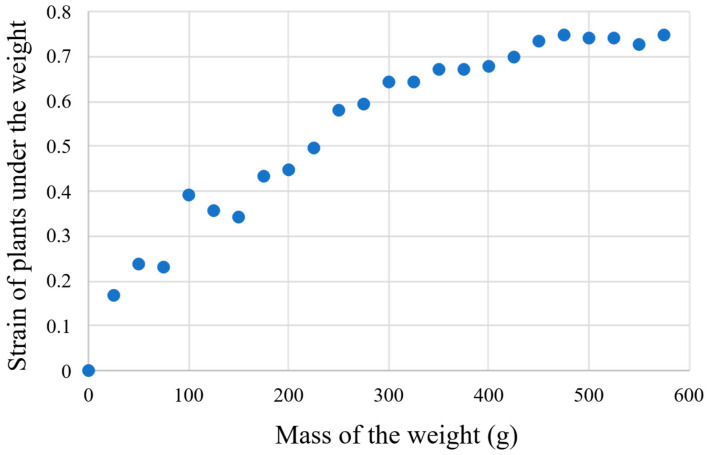
The scatterplot showing the load-strain behavior of plant cross-linked structures.

In addition, we decided to investigate and quantitatively evaluate the load-strain behavior of the cross-linked plant structures based on the deformation of the structures when weights were placed on them. Here, we defined the strain as the amount of change in the apparent thickness of the central portion of the plant cross-linked structure on which the weights were placed. For this thickness, we calculated it from photographs taken throughout the load-bearing experiments, using the size of the experimental device as a guide. As a result, as shown in Figure 6, we obtained a scatter plot showing the load-strain behavior of the plant cross-linked structure. The horizontal axis of this graph represents the mass of the weight placed on the plant cross-linked structure, and the vertical axis represents the strain when the weight was placed on the central portion of the plant cross-linked structure. Sampling was carried out at 25 g intervals. From Figure 6, it can be seen that, in the low mass range of the weights, up to about 200 g, the amount of strain change was large due to the wide gap between the plants, and the amount of strain change became smaller as the mass increased in the latter half of the range. Therefore, we can infer that, up to a mass of about 200 g, the plants located mainly at the top of the bridge contribute to supporting the weight, but as the mass increases, the entire bridging plant bundles together to support the weight.

## 5. Bridge-Crossing Experiment Using a Robot

**Figure 7:**
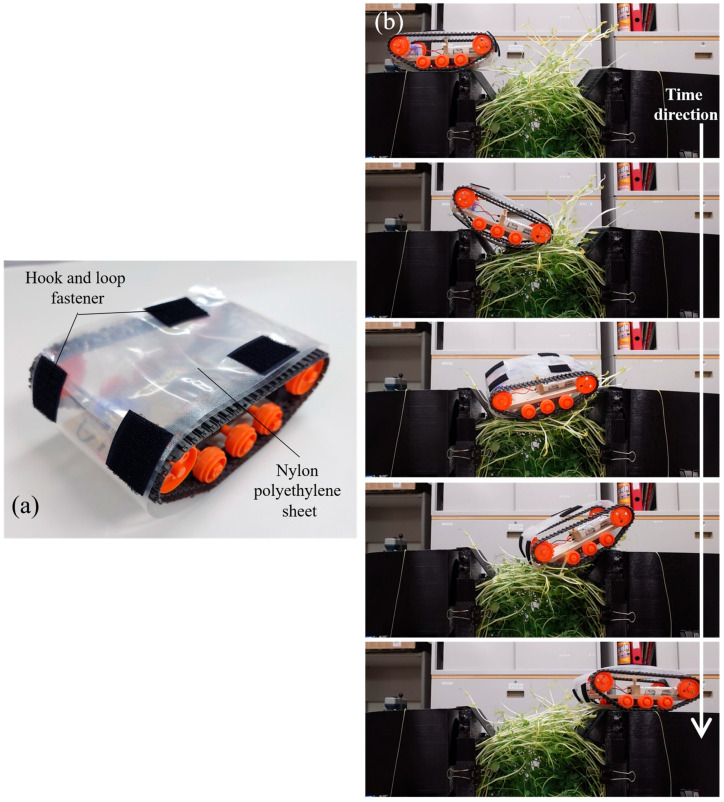
The experiment of a robot crossing over the bridged plants. (**a**) The appearance of the robot used in the experiment. It has been improved to make it easier to move on the plant. (**b**) Robot moving over cross-linked plants. From top to bottom, the images are lined up with the time since the start of the robot moving.

The experiment showed that the device cross-linked by plant growth can place objects weighing about 575 g. Therefore, we decided to conduct an experiment to simulate the actual use of plants connecting two separated sections. Here, a tank-type robot (Tamiya, Inc., Shizuoka, Japan) was used to perform the movement experiment on the cross-linked plants (Figure 7a). The robot is 171 mm long, 105 mm wide, and 56 mm tall. The speed of the robot on flat ground was about 1.72 cm/s. Cross-linked plants are actually sparse in their structure. Therefore, in this experiment, we considered the possibility that the robot would be stuck between plants if a wheel-driven robot or a small robot was used. Based on the above, we chose a tank-type robot and covered it from the outside with a nylon polyethylene sheet. This prevented the crawler from entrapping the plant and increased the robot’s contact surface with the plant. The robot weighed 210 g, including batteries and other components.

The equipment setup for this test was the same as in Section 4. The growing period was 29 days. The average temperature and humidity of the growing environment during this period were 19.1 °C and 73.6%, respectively. The distance between devices was set at approximately 20 cm. In this experiment, we installed a ramp on top of the device for the robot to climb from the bridged plant to the top of the device. In addition, we attached hook and loop fasteners to the robot’s surface and ramp to increase friction for the robot’s climbing.

Figure 7b shows the bridge-crossing experiment using the robot. It shows that it is possible for the robot to descend from the device onto the cross-linked plant and then cross over to the other side of the device. We attempted a total of 10 bridge crossings using the robot. Of these, there were seven successes and three failures. We considered the following as causes of failure. One is a fall due to the fact that the width of the robot and the width of the bridged plant were about the same. The second is due to the fact that the robot was unable to climb the ramp installed on the device. Therefore, it is expected that by improving the shape of the device, it can be used more stably for robot bridge crossing in the future.

In addition, we estimated how many times this robot can cross over the plant bridging structure in terms of its durability. Here, from Section 4, we referred to the number of times a weight of more than 210 g was placed. In that case, we confirmed that the structure did not rupture, even when we placed a weight of more than 210 g approximately 70 times. Therefore, it is expected that the robot used in this experiment can attempt to cross over the plant cross-linking structure at least 70 times.

## 6. Discussion

In this study, we proposed the concept of a plant bridge and conducted an experiment to connect two separated devices using plant growth. Plants grew from each device toward each other’s light source, connected by the tangle and friction of plant stems or leaves. The plant connection was strong enough not to be destroyed when a weight of over 500 g was placed near the center of the cross-linked area. The cross-linked plants were slightly bent by the weights, but since the weights were supported at the top of the device, we assume that the plants are resistant to further loading. In addition, this result indicates that the cross-linked plant structure has a load-bearing capacity of more than 6.6 times greater by the mass ratio. We also indicated that the robot can cross between the devices over this bridged plant.

The direction of plant growth was encouraged to the light source mounted on the device on the opposite side of the planted side. However, not all of the plants on the device grew in the intended direction. Most of the plants planted on the upper side of the device’s light source irradiation port were growing toward the other device’s light source in an arch shape. However, many of the plants planted on the lower side of the light source were observed to be growing toward the light source on their own side, rather than toward the light source on the other side. This blocked light and inhibited growth in a spindly way on the other side, causing plants to grow tall in areas where light did not reach. Therefore, we consider it necessary to improve the position of the light source and sowing position in the future. Also, we would like to examine models of plant bridging structures that assume in advance the uneven growth of plants.

In this study, we investigated the load-bearing capacity of cross-linked structures made of plants by placing weights on them. However, we were unable to gain insight into the collapse of this structure. This is because the loading procedure was not well-defined due to the particular characteristics of our proposed structure. Therefore, in the future, we intend to develop a more standardized loading procedure and conduct experiments leading to the collapse of plant cross-linked structures. Through this, we would like to conduct research on the collapse mechanism of this structure by investigating its resistance to higher loads and its behavior under longer-term pressure.

In this study, experiments were conducted only in the range of approximately 15 to 20 cm and 30 cm between the devices of the plant cross-linking equipment. In particular, we have confirmed that plants connect successfully at 15 to 20 cm; although, we have not yet been able to conduct experiments at shorter or wider ranges. Therefore, in the future, we would like to conduct experiments by changing the distance between the devices in a more varied range and investigate the effect of the distance between the devices on the strength of the plant cross-linked structures.

In this study, the experimental equipment was set up in a darkened, ventilated room, and plants were grown in it. In this process, the temperature and humidity were not specially controlled, so the average temperature and humidity differ depending on the experimental period. However, especially in terms of load capacity, it does not appear that the temperature and humidity range of the experimental environment has a significant effect. However, we think that it is necessary to investigate how much the degree of plant growth changes and how it affects the strength of the structure when it is used in a more natural environment, such as outdoors. Therefore, we intend to conduct experiments under more extreme temperature and humidity conditions and to select plants that can grow in extreme environments. In addition, we will also examine ways to counter changes in temperature and humidity by improving the equipment, for example, by controlling the water temperature inside the equipment.

In addition, in this experiment, plant growth was conducted in an environment with sufficient water, and water was constantly delivered to the medium throughout the experiment. However, under environments where the moisture content of the medium is not constantly controlled, plants may die due to a lack of moisture. Plants that have withered have their biophysical characteristics greatly changed. Therefore, in the future, it is necessary to conduct more practical experiments assuming that water cannot be supplied at all times. We plan to investigate changes in the ability of plants to self-heal, change shape, and increase rigidity when given different amounts of water and to determine the amount of water required for plant composite materials. Also, we intend to investigate the physical characteristics of the plant after withering, assuming that continued plant growth is not required. In addition, in order to utilize continuous plant growth, we would like to select plants that can be used for longer periods of time in more arid and other natural environments and to explore methods for dynamic water recovery.

Future prospects include incorporating the concept of compartmentalization. This is a protective strategy of sacrificing body parts under high-load conditions observed in plants and animals, such as lizards and oxalis [30,31,32]. Although we have not yet observed such a property in the pea that we are currently using, we think that the ability to perform passive self-cutting under high loads is significant for improving the robustness of the equipment. Therefore, in the future, we would like to consider the use of plants with the concept of compartmentalization and the adoption of monitoring technology through the improvement of equipment.

As for future applications, due to the time constraints on plant growth, it is expected to be used in relatively large structures, such as buildings. We also consider that it is possible to strengthen or deform a structure by controlling the growth of plants. This is one of the relatively low environmental impact methods using natural materials. In addition, for dynamic use, we expect to use the system for longer-term tasks in environments that are out of reach of humans.

## Data Availability

Data is contained within the article.

## Abbreviations

The following abbreviations are used in this manuscript:

PLA	Poly-lactic acid
LED	Light-emitting diode
